# Urethral obstruction from dislodged bladder Diverticulum stones: a case report

**DOI:** 10.1186/1471-2490-12-31

**Published:** 2012-11-08

**Authors:** Linus I Okeke, Augustine O Takure, Sikiru A Adebayo, Olukayode Y Oluyemi, Abimbola AA Oyelekan

**Affiliations:** 1 Division of Urology, Department of Surgery, University College Hospital, and College of Medicine, University of Ibadan, Ibadan, Nigeria

**Keywords:** Urethral obstruction, Diverticulum stones, Urinary retention

## Abstract

**Background:**

Secondary urethral stone although rare, commonly arises from the kidneys, bladder or are seen in patients with urethral stricture. These stones are either found in the posterior or anterior urethra and do result in acute urinary retention. We report urethral obstruction from dislodged bladder diverticulum stones. This to our knowledge is the first report from Nigeria and in English literature.

**Case presentation:**

A 69 year old, male, Nigerian with clinical and radiological features of acute urinary retention, benign prostate enlargement and bladder diverticulum. He had a transurethral resection of the prostate (TURP) and was lost to follow up. He re-presented with retained urethral catheter of 4months duration. The catheter was removed but attempt at re-passing the catheter failed and a suprapubic cystostomy was performed. Clinical examination and plain radiograph of the penis confirmed anterior and posterior urethral stones. He had meatotomy and antegrade manual stone extraction with no urethra injury.

**Conclusions:**

Urethral obstruction can result from inadequate treatment of patient with benign prostate enlargement and bladder diverticulum stones. Surgeons in resource limited environment should be conversant with transurethral resection of the prostate and cystolithotripsy or open prostatectomy and diverticulectomy.

## Background

The incidence of urolithiasis has increased in Germany, but the situation is variable in the United States [1]. The northern part of Nigeria has a higher incidence of stone disease and the majorities are located in the upper urinary tract [2]. However, the urinary tracts stone distribution in Lagos was renal stones 22.2%, ureteric 35.5%, and vesical was 35.5%. While urethral stones constituted 2.2% [3] and this percentage of urethral stone is similar to reports from the developed countries [4]. Urethral stone frequently originated from the upper urinary tract or the urinary bladder [5] and was commonly found in either the anterior or posterior urethra where it often presented with acute urinary retention. Primary urethral stones are rare, but secondary stones are seen in patient with urethral stricture [6]. We present urethral obstruction from dislodged bladder diverticulum stones and its management in a resource limited environment. This to our knowledge is the first case in Nigeria and in English literature search.

## Case presentation

A 69 year old, male, Nigerian who was initially seen with a 4 year history of storage and voiding lower urinary tract symptoms. At that presentation, he had an episode of acute urinary retention that was relieved by urethral catheterization. Thereafter, he had a failed trial of voiding without catheter and was advised to change his catheter at 3 weekly intervals while on α-adrenergic receptor blocker. The digital rectal examination revealed benign prostate enlargement.

The abdomino-pelvic ultrasound showed a 64gm prostate and a posterior bladder diverticulum that measured 8x8x7cm^3^. The serum prostate specific antigen was 7.5ng/dl and prostate biopsy was reported as nodular hyperplasia and chronic prostatitis while the serum electrolytes, urea and creatinine were normal. He was treated for chronic prostatitis and continued to take the α-adrenergic receptor blocker. He was lost to follow up for 4 years during which time he had a TURP elsewhere and remained symptom free.

He presented to us with retained urethral catheter that was passed 4months prior to seeing us for acute urinary retention. After removal of his urethral catheter, it was impossible to pass another one therefore a suprapubic cystostomy was performed. Urodynamic studies were not done.

**Figure 1:**
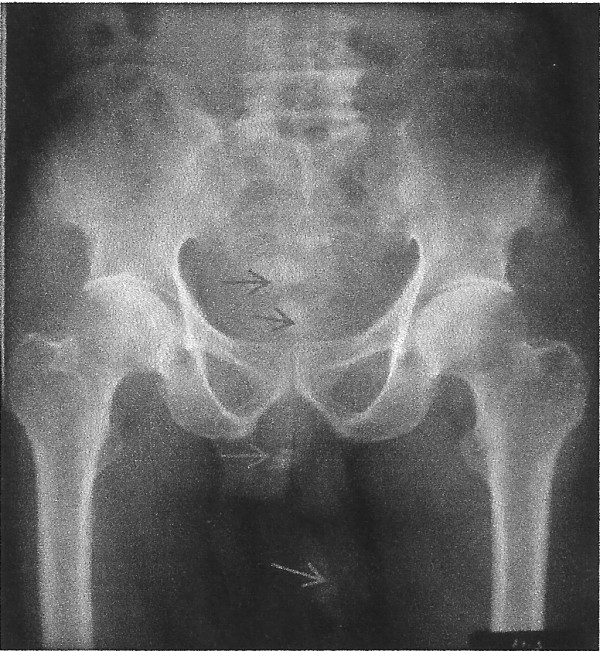
Plain radiograph showing diverticulum, bladder neck, and urethral stones (arrow).

Four weeks after the suprapubic cystostomy, he presented with severe urethral pain and examination revealed impacted urethral stone at the tip of his external urethral meatus and completely granular anterior urethra. Plain radiograph of the penis and lower abdomen showed radio-opaque shadows in the bladder, bladder diverticulum, posterior and anterior urethra (Figure 1). The clinical diagnosis was urethral obstruction from dislodged bladder diverticulum stones.

**Figure 2:**
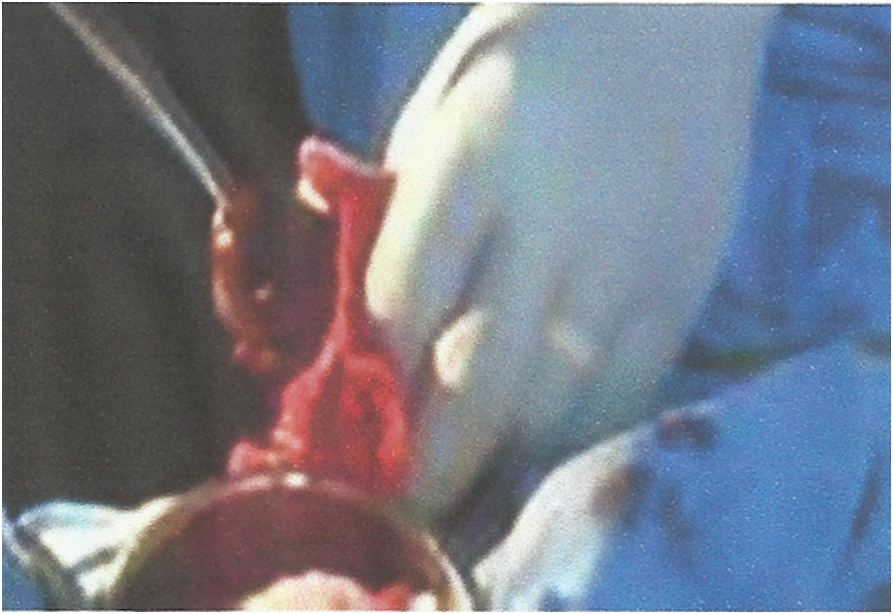
Manual antegrade removal of urethra stones.

**Figure 3:**
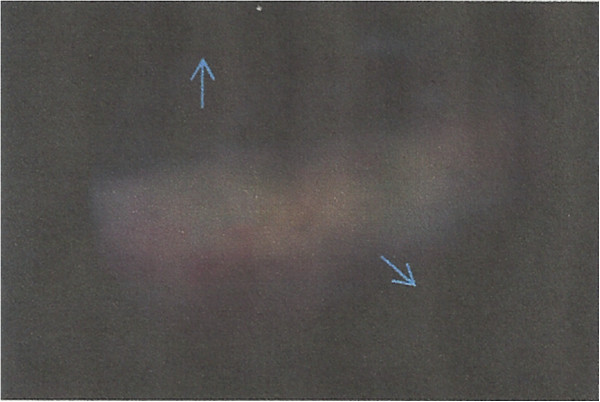
bladder mucosa bridge separating bladder cavity (arrow up) and posterior bladder diverticulum below (arrow down).

Under caudal anaesthesia (2% plain xylocaine solution) and sedation with intramuscular pentazocine 30mg, we attempted to push the stones endoscopically into the bladder but this failed. Thereafter, he had meatotomy with 2% xylocaine jelly being instilled into the urethra. After waiting for 10 minutes, he had antegrade manual stone extraction with sinus forceps and intermittent lubrication of the urethral with 2% xylocaine jelly (Figure 2). All the stones were completely removed and a check Cystoscopy confirmed the wide neck urethral Diverticulum (Figure 3).

## Discussion

Primary urethral stones are a rare cause of acute urinary retention [1,2]. In this patient, long standing benign prostate enlargement predisposed him to the development of the bladder diverticulum. Any cause of bladder outlet obstruction such as neurogenic bladder [3], posterior urethral valves, benign prostate enlargement, or urethra stricture [3] may be complicated by bladder or urethral stones as seen in this patient. Our patient presented with acute urinary retention as well as difficulty with urethral catheterization. Prolonged urethral catheter leads to formation of encrustations around the catheter balloon resulting in catheter retention. Similarly, the prolonged lower urinary tract symptoms in this patient may account for the bladder diverticulum and diverticular stones prior to the TURP. The sudden bladder decompression following the catheter removal could be responsible for the stones being driven down the urethra.

During the TURP the diverticulum mouth was widened but the stones could not be extracted probably due to their sizes. Supra pubic cystolithotripsy [6] then followed by TURP or simultaneous suprapubic cystolithotripsy and TURP is preferred. In the developing country with limited endoscopic equipment, open retropubic or transvesical prostatectomy, diverticulectomy and stone removal is an alternative treatment. This may reduce the patient’s agony.

Imaging studies often localize these stones commonly in the posterior urethra [6-9] or anterior urethra [2] though at times computed tomography may not be able to identify impacted urethral stone [5]. In our patient the plain radiograph of the pelvis and male external genitalia confirmed the anterior and posterior urethral stones (steinstrasse).

Other reports and studies have demonstrated that urethral stones could be easily pushed back either manually with a catheter or endoscopically into the urinary bladder [2,6,8] This was not our experience, as the impacted urethral stones was not pushed back, hence meatotomy, antegrade manual extraction, generous urethral lubrication with 2% xylocaine jelly was performed. Urethral stones that are pushed into the urinary bladder are removed by open cystolithotomy in the tropics where facilities are not available [2]. While in the industrialized countries, extra corporeal shock wave lithotripsy [8] or cystolithotripsy [6] are effective and safe options of treatment. Electrohydraulic endourethral lithotripsy is quite good for accessible urethral stones and is least traumatic [7].

Urethro-cystoscopy confirmed complete stone removal with no urethral injury.

## Conclusions

In adequate treatment of patient with benign prostate enlargement and bladder diverticulum stones result in anterior and posterior urethral stones manifesting as urethral pain and acute urinary retention. This can safely be treated by antegrade manual stone extraction under caudal anesthesia in resource limited environment. The preferred treatment is TURP with cystolithotripsy at the same sitting.

## Consent

Written informed consent was obtained from the patient for publication of this case report.

## Competing interests

The authors declare that they have no competing interests.

## Authors’ contributions

LIO; participated in the concept, helped in the critical review of all drafts, read and approved the final manuscript. AOT; participated in the concept, participated in the design, initial drafts and critical review of all the drafts, literature review and updates of literature, and the final manuscript. SAA participated in the review of drafts and the final write up. OYO; patient investigations, administered the caudal anaesthesia block to the patient, preparation for procedure, photography and participated in the initial drafts of the manuscript. AAAO; administered the caudal anaesthesia to the patients, and initial drafts. All authors read and approved the final manuscript.

## Pre-publication history

The pre-publication history for this paper can be accessed here:

http://www.biomedcentral.com/1471-2490/12/31/prepub
